# Gastric Volume Changes in Preterm Neonates during Intermittent and Continuous Feeding-GRV and Feeding Mode in Preterm Neonates

**DOI:** 10.3390/children8040300

**Published:** 2021-04-15

**Authors:** Rozeta Sokou, Ioanna N. Grivea, Eleni Gounari, Polytimi Panagiotounakou, Maria Baltogianni, George Antonogeorgos, Fedra Kokori, Aikaterini Konstantinidi, Antonios K. Gounaris

**Affiliations:** 1 Neonatal Clinic-NICU, General Hospital of Nikaia “Agios Panteleimon”, 3 D. Mantouvalou Str., 18454 Piraeus, Greece; ppppolytimi04@gmail.com (P.P.); gantonogeorgos@gmail.com (G.A.); kmaronia@gmail.com (A.K.); 2 Neonatal Clinic-NICU, University General Hospital of Larissa, 41222 Larissa, Greece; ioanna.grivea@gmail.com (I.N.G.); angounaris@med.uth.gr (A.K.G.); 3 Royal Alexandra Children’s Hospital Brighton, Eastern Road, Brighton BN2 5BE, UK; elenigounari@gmail.com; 4 Neonatal Clinic-NICU, University General Hospital of Ioannina, 45110 Ioannina, Greece; mbalt@doctors.org.uk; 5 Department of Radiology, General Hospital “Agios Panteleimon”, 18454 Piraeus, Greece; gkandilis@yahoo.gr

**Keywords:** continuous feeding, feeding intolerance, gastric emptying time, gastric residual volume, intermittent feeding, very low birth weight newborns

## Abstract

**Background:** We aimed to evaluate gastric volume changes during intermittent milk feeds (IMF) and continuous milk feeds (CMF) in very premature neonates (VPN), with gastric residual volume (GRV) based on antral cross-sectional area (ACSA) measurements and to examine if there were differences in GRV between the two feeding methods. **Methods:** A randomized prospective clinical trial with crossover design was conducted in 31 preterm neonates (gestational age < 30 weeks). Gastric volume was assessed twice in each neonate (during IMF and CMF feeding), at 7 specific time points during a 2-h observation period by measuring ACSA changes via the ultrasound (U/S) method. **Results:** There was a significantly different pattern of gastric volume changes between the two feeding methods. GRV, expressed as the median percentage of ACSA measurement at 120 min relative to the higher ACSA measurement during IMF, was found to be 3% (range 0–25%) for IMF and 50% (range 15–80%) for CMF. Neonates fed with IMF had a shorter mean gastric emptying time compared to those fed with CMF (*p* = 0.0032). No signs of feeding intolerance were recorded in either group during the period of observation. **Conclusions***:* Our results showed that gastric volume changes and gastric emptying time in VPN, based on ACSA measurement changes, depend on the milk feeding method. No gastrointestinal complications/adverse events were noted with GRV up to 80% with CMF.

## 1. Introduction

Feeding intolerance, defined as the inefficiency of the gastrointestinal system of very preterm neonates (VPN) to digest milk, is associated with the presence of increased pre-feed gastric residual volumes (GRVs) and expressed with vomiting/abdominal distention/desaturations/bradycardia/apnea, often leading to the disruption of a feeding plan in this fragile population.

During the first weeks of life, very low birth weight (VLBW) and especially extremely low birth weight (ELBW) neonates, commonly exhibit feeding intolerance and a delay in gastric emptying [1,2,3]. Gastric emptying can act as an indicator of feeding tolerance and it is assessed by measuring the quantity and the quality of aspirated GRV. Gastric emptying time in VPN depends on the volume and type of milk and is independent of postnatal age [4]. Several factors such as milk type (breast milk or formula), use of breast milk fortifier, infusion rate, body position and feeding mode (intermitted-bolus or continuous) influence gastric emptying in preterm neonates [5,6]. The most appropriate feeding method (intermittent or continuous) for preterm neonates to achieve full enteral feeding remains an issue of controversy.

A meta-analysis conducted by Premji et al. [7] showcased the difficulties in making universal recommendations regarding the best feeding method for preterm infants with a birth weight less than 1500 g.

There is currently ongoing research on increased GRV in intermittently fed preterm neonates and the incidence of necrotizing enterocolitis (NEC). There are controversies regarding the correlation between increased GRV and NEC in preterm neonates, as the relevant data are insufficient [8,9,10,11,12]. Several methods of assessing the GRV have been trialed in preterm neonates such as the gastric aspiration technique via gastric tube and imaging techniques, but wide variations exist as to what constitutes significant GRV. The ultrasound (U/S) method has been used successfully to investigate gastric emptying in preterm infants [13]. Perrella SL et al. [14] showed that there was a significant relation between antral cross-sectional area (ACSA) and the proportion of feed volume delivered (*p* < 0.001). Furthermore, this method presents a more physiological and non-invasive technique [15].

Limited data are available from trials which have compared bolus versus continuous feeding and the existing evidence is insufficient to determine an optimal feeding strategy. Yan Wang et al. [16], in a meta-analysis of randomized controlled trials have found that intermittent feeding may be more beneficial for low birth weight infants, but well-designed studies and evidenced-based clinical practice are required to determine the most appropriate feeding method for premature infants with low birth weight.

Although in two different publications [17,18] the same upper threshold levels of GRV were applied for determining feeding intolerance during both continuous and intermittent methods of feeding, there are still controversies regarding what constitutes “normal GRV” in VPN fed by continuous feeding.

The aim of our study was to record and evaluate gastric volume changes during intermittent and continuous method of feeding in VPN and to assess GRV based on ACSA measurements, as well as to examine if there are differences in GRV between the two feeding methods. Our secondary aim was to compare gastric emptying time between intermittent and continuous feeding mode, as well as gastrointestinal complications of VPN fed with the two feeding methods.

## 2. Materials and Methods

This single-center, randomized, prospective crossover clinical trial was conducted at the Neonatal Intensive Care Unit (NICU) of Agios Panteleimon Hospital, over a period of one year. The Institutional Review Board approved the protocol (18 May 2016, 29/2), which was designed, conducted, and reported in compliance with the Declaration of Helsinki. Parental informed consent was obtained prior to recruitment.

### 2.1. Participants

**Figure 1:**
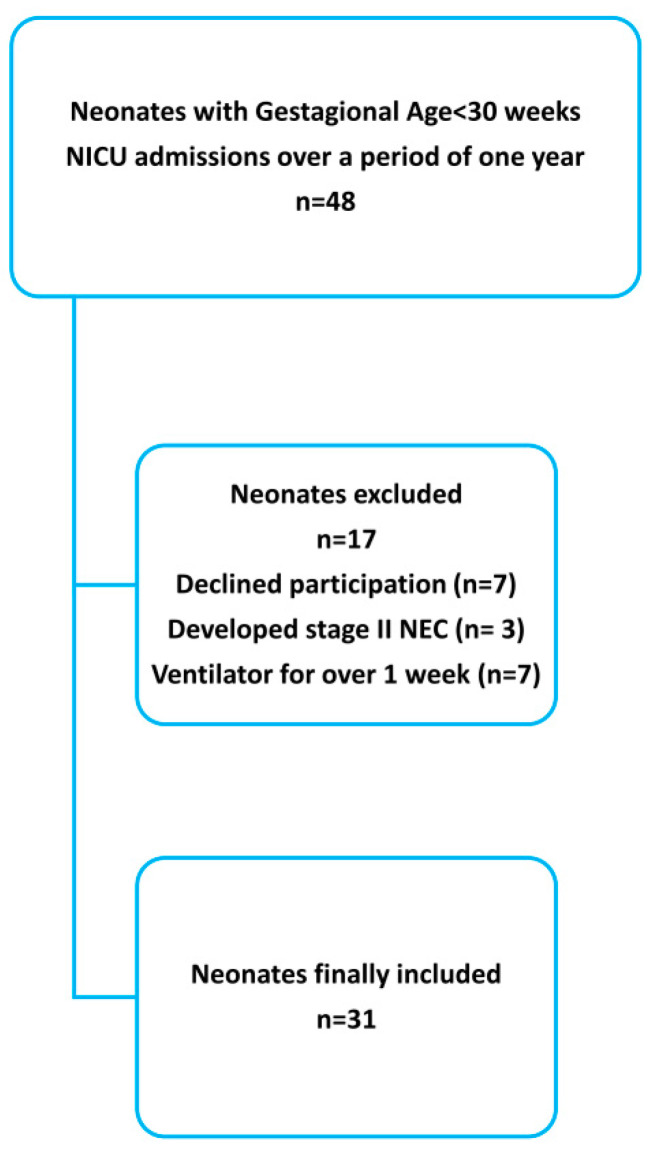
Flow chart of study population.

The study population consisted of preterm neonates with a gestational age (GA) < 30 weeks, hospitalized in the NICU. Exclusion criteria included severe congenital abnormalities, ventilator dependence beyond the first week of life, as well as stage II or III NEC according to the modified Bell criteria [19]. Recruitment procedure data are presented in the flow chart (Figure 1).

### 2.2. Study Protocol

The 31 newborn babies included in the study were randomly assigned to be fed with one of the two feeding methods, either intermittent-bolus (group A) or continuous (group B) and underwent serial ultrasound measuring of ACSA changes during a 2-h observation period. Sample size calculation was performed prior to study design in order to compute the sufficient study sample size. The study was powered at 80% to detect significant standardized effect size differences of 0.25 between two factors in repeated measurements of analysis of variance, under the assumption of a correlation coefficient between measurements r = 0.5 at a significance level a = 0.05.

During continuous feeding, neonates received the daily milk volume by orogastric tube (OGT) with a constant rate for 24 h via a pump. During intermittent-bolus feeding, neonates received OGT feeds via gravity for less than 10 min every 2 h. The pre-feed assessments of GRV were performed through aspiration before every second feed by the responsible nurse. In our NICU, VPN were comfortably contained within the nests in the supine, prone or right lateral position interchangeably. Neonates were fed in the supine position.

All neonates were enrolled in the study within the first week of life. Randomization for the crossover design was achieved with the use of sealed opaque envelopes. The simplest model AB/BA was used for randomization. Subjects allocated to the AB study arm received intervention A first, followed by intervention B, and vice versa in the BA arm. All study neonates received both modes of feeding and they acted as controls of themselves. The observation period started when the neonates were receiving enteral milk volume of more than 80% of their daily fluid intake. Sixteen neonates were initially assigned to the bolus feeding group and subsequently to the continuous feeding group, while the remaining 15 were assigned vice versa. Before each measurement, the neonates had been on the same feeding regimen (type and milk volume) for at least 48 h. In all cases, there was a minimum of three days washout interval between the two measurements to eliminate any carry-over effects, but sometimes this period was extended up to five days depending on the availability of the radiologists

According to the NICU protocol, in VPN enteral feeding was initiated on the 1st–2nd day of life, depending on their clinical status. Minimal enteral feeding was initially introduced at 1 mL and up to 2 mL every 2 h for about three days, depending on the neonate’s level of care. Bolus feeding through OGT via gravity was the standard method of feeding in our NICU. The responsible nurse placed the OGT after taking a measurement of the distance from the nose to the ear and the end of the sternum, with the addition of 1 cm and the correct placement was checked with the aspiration of gastric content. X-rays obtained for other clinical reasons helped with the final assessment of gastric tube placement. Following minimal feeding, standard milk increments of about 20–30 mL/kg/day were administered every 2 h until full enteral feeding was reached (at a volume of 200–210 mL/kg/day) in an effort to achieve a weight gain of >15–20 g/day.

Every neonate received the same type of milk (breast milk with fortifier or a preterm formula containing 81 kcal/100 mL) during the 48-h period before each measurement. The median milk quantity administered to each newborn during the procedure was 17 mL/2 h (range 9–22) and this amount remained the same in both measurements.

It is worth noting that each newborn was on the same respiratory support during both assessments; either on bubble nasal continuous positive airway pressure (nCPAP) with pressure up to 7 cm H_2_O, or oxygen via incubator with FiO_2_ < 30%, or room air. During the study period, no neonate received caffeine and nCPAP was used for apnea treatment. Signs of feeding intolerance and any gastrointestinal complications presenting during the observation period which included the day of each measurement and a 48-h period before that, were recorded in detail by the nursing staff caring for each neonate.

### 2.3. Ultrasound Examination

The ultrasound method proposed by Newell et al. [20], was used to evaluate gastric volume changes. After the 10 a.m. morning feed, a radiologist tracked and measured the changes of ACSA using U/S equipment. Measurements were obtained over the next 2 h; every 10 min for the first 30 min and thereafter every 30 min; resulting in a total of 7 measurements. Transverse sections of the pyloric antrum were taken, with the vertebral column and origin of the superior mesenteric artery serving as guidelines. The measurements were performed with the newborn lying in a right lateral position. In neonates fed intermittently, gastric residual volume was assessed through aspiration and was thrown away before the measurement procedure was started to ensure that any residual of the previous feeding would not affect the following ACSA measurement. The residual volume was less than 20% of the previous feeding volume in all of the intermittently fed neonates. The first measurement of the ACSA was conducted immediately after the milk feed administration. In continuously fed neonates, the radiologist measured the ACSA during continuous milk administration via pump, from 10 a.m. and for 2 h at the same 7 time-specific points.

The ultrasonographic method involves minimal handling; the probe is in touch with the baby’s abdomen only for few seconds in every measurement (less than 2 min are needed in total for the 7 measurements) and the whole examination can be performed in the cot. All measurements were taken with the Easote My Lab 50 Ultrasound machine and a 7.5 MHz probe was used. The same radiologist, performed all U/S measurements.

### 2.4. Outcome Measures

ACSA measurements according to Newell’s method [20] were recorded at 7 time-specific points for each neonate during the two feeding methods. Gastric content changes were evaluated based on these serial ACSA measurements. GRV was expressed as a percentage of the ACSA measurement at 120 min relative to the higher ACSA measurement during intermittent feeding.

Gastric emptying time was defined as the time needed for the ACSA measurement (in cm^2^) to reach half of its higher value during intermittent feeding for all study neonates (as they were acting as controls of themselves).

Data on demographics, maternal medication during pregnancy, neonatal physiological parameters, and clinical findings were recorded. Small for gestational age (SGA), neonatal respiratory distress syndrome (RDS), NEC and bronchopulmonary dysplasia (BPD, the need for continuous supplemental oxygen at 28 days of age), defined according to the literature [19,21,22,23], were also recorded. Regurgitation, vomiting, bile or blood stained aspirates, visible bowel loops, abdominal distension and heme-positive stools, were considered as possible gastrointestinal complications. In neonates fed intermittently, gastric residue of more than 50% of the previous feeding volume was also considered as a possible gastrointestinal complication.

### 2.5. Statistical Analysis

The normality of the distribution of the variables was evaluated using statistical tests and diagnostic graphs (Shapiro–Wilks and Kolmogorrov–Smironv tests of normality and P-P plots and Q-Q plots, accordingly). Continuous variables are presented as mean and standard deviation if they are normally distributed or median and 1st and 3rd quartile if their distribution is skewed. Categorical variables are presented as absolute and relative frequencies. The Wilcoxon matched paired rank test was used in order to assess differences in ACSA between bolus and continuous fed VLBW infants at each time point. Repeated measured analysis of variance (repeated ANOVA) was applied, after a log-transformation of ACSA values for complying with the assumption of normality, in order to assess the effect of the feeding method (bolus vs. continuous) in the changes of ACSA over time, adjusting for the possible confounding effect of gender, ventilation status (nCPAP) and birth weight in all the different time points. In order to address the *p*-value inflation due to multiple statistical comparisons, the Bonferroni correction was applied. All reported probability values (*p*-values) are based on two sided tests and compared to a significance level of 5%. SPSS 18.0 software was used for the all calculations (SPSS Inc. Released 2009. PASW Statistics for Windows, Version 18.0. SPSS Inc., Chicago, IL, USA).

## 3. Results

The clinical characteristics of the study population are presented in Table 1.

The first measurements were performed on the 15th (median, range 11–32) day of life and the second ones on the 19th (median, range 14–35) day of life.

**Figure 2:**
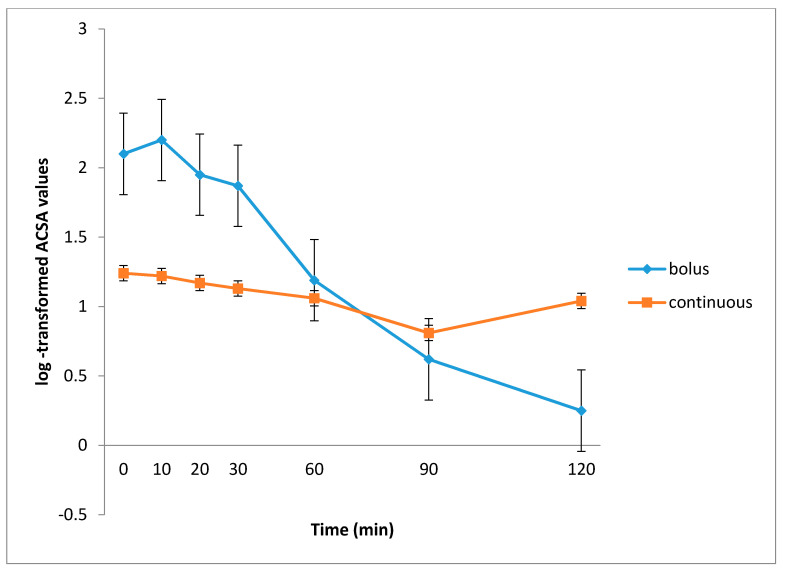
Pattern of gastric antral cross-sectional area (ACSA) values (log-transformed) reduction through different study time points according to feeding method (intermittent vs. continuous).

The mean log-transformed ACSA values for continuously fed newborns were significantly lower at 0, 10, 20 and 30 min (*p* < 0.001) and significantly higher at 90 and 120 min (*p* < 0.001) compared to the respective ACSA values for intermittently fed neonates. At 60 min the ACSA values were not significantly different between the two groups. (Table 2, Figure 2).

Error bars represent standard error of the mean.

Moreover, the variability in ACSA values at each time point, expressed as an interquartile range (3rd quartile value minus 1st quartile) were assessed and found to be very low during the continuous feeding method (interquartile range varying from 1.0 to 1.45 cm^2^) while during the intermittent feeding method they were very high (interquartile range varying from 0.2 to 2.2 cm^2^). (Table 2). In the U/S measurement at 120 min median ACSA values were 0.1 cm^2^ and 0.8 cm^2^ for intermittently and continuously fed neonates, respectively, and this difference was statistically highly significant (*p* < 0.001).

GRV, expressed as the percentage of the last ACSA measurement (at 120 min) relative to the higher ACSA level, was found to be 50% (median, range 15–80%) during continuous feeding. It is noteworthy that although 13 out of the 31 neonates had GRV values between 50–80% with continuous feeding, no gastrointestinal complications were recorded. For intermittent feeding, GRV value was 3% (median, range 0–25%).

There was a statistically significant difference in the mean gastric emptying time between the intermittently fed (group A) and continuously fed (group B) neonates [37.8 ± 15 min vs. 57.7 ± 29 min (*p* = 0.0032)]. For neonates fed continuously, gastric emptying time was not calculated in all cases, as 11 out of 31 neonates’ ACSA measurement did not reach half of the higher value.

During the observation period, no infant displayed signs of feeding intolerance such as vomiting, apnea, bile/blood stained aspirate, visible bowel loops, abdominal distension and no infant developed NEC.

## 4. Discussion

This single-center, randomized, prospective crossover clinical trial showed that in very preterm neonates, the milk feeding method had an influence on gastric volume changes as well as on gastric emptying time, based on ACSA measurement changes. Moreover, no gastrointestinal complications/adverse events were noted when GRV was up to 80% in continuously fed VPN.

There is an ongoing debate among neonatologists throughout the years regarding the optimal timing, quantity, and method for feeding VLBW neonates. One meta-analysis concluded that developing universal recommendations regarding the best feeding method for infants less than 1500 gr is problematic [7] and more recently Wang et al. [16] reported that intermittent feeding may be more beneficial for low birth weight neonates, but well-designed and evidenced-based studies are required to determine the most appropriate feeding method for preterm neonates.

Continuous enteral feeding has been shown to help limit the risk of hypoxic-ischemic gut damage in critically ill preterm neonates [24]. Continuous feeding also appears to maintain the gastrointestinal hormones, such as gastrin and insulin, at a high level increasing the absorption, reducing energy expenditure [16] and increasing duodenal motility, while bolus feeding decreases it [25]. Dani C. et al. [26], have demonstrated that bolus milk feeding increases splanchnic oxygenation in both appropriate for gestational age (AGA) and SGA infants, whereas continuous feeding does not.

Extensive research has been conducted by neonatologists on what exactly constitutes an “acceptable” or “normal” level of GRV following an intermittent feeding regime [27,28,29] but controversies persist as to what constitutes a “normal” GRV during continuous feeding, which would permit the uneventful continuation of enteral feeding of preterm neonates. Dollberg S. et al. [17], considering the same GRV upper threshold of <40% as acceptable for both intermittent and continuous feeding methods, came to the conclusion that the intermittent feeding method is more effective than continuous in improving feeding tolerance in small VLBW infants. Dsilna A. et al. [18] also adopted similar upper thresholds (<50%) as indicators of feeding tolerance for these two feeding methods, and found that continuous feeding seems to be better than intermittent feeding with regards to gastrointestinal tolerance and growth of very preterm neonates. On the other hand, Rovekamp-Abels LW et al. [30], in a recent study on bolus and semi-continuous feeding methods in very preterm neonates, evaluated using a larger gastric residue prior to postponing feeding and concluded that bolus and continuous feeding are equally suitable strategies for these neonates.

Despite the wide variation regarding the definition of an abnormal GRV, the most cited GRV considered as abnormal is the one that exceeds the hourly infusion rate or 50% of milk administered every two hours [12]. In our study upper GRV thresholds during bolus feeding were similar to those already reported. For the first time in the literature, we found GRV up to 80% to be well tolerated during continuous feeding. Thus, our results could lead to the consideration of a higher upper threshold for GRV during continuous feeding and might help re-evaluate the current knowledge base.

The usefulness of the systematic evaluation of GRV in VPN is the subject of a long-standing debate among neonatologists. Some authors propose omitting the routine evaluation of pre-feed GRV during bolus feeding [12,31] and more recently others [32], suggest that the omission of gastric residual evaluation in VPN should translate into evidence-based practice, as it increases the delivery of enteral nutrition and improves weight gain leading to earlier hospital discharge. On the other hand, a recent meta-analysis came to the conclusion that there is low quality evidence to suggest that routine monitoring of GRV increases the risk of feed interruption episodes as well as the time to reach full enteral feeding [10].

High GRV is a sign of feeding intolerance and has been used as an early marker to prevent NEC. Both Cobb B.A. et al. [8] and Bertino E. et al. [9] found a correlation between high GRV and NEC. The aforementioned meta-analysis [10] concluded that there were insufficient data to support or not GRV evaluation for the prevention of NEC. On the contrary, Parker L.A. et al. [32] supported the omission of gastric residual evaluation in extremely preterm neonates, although the study was not directed to evaluate the risk of NEC In this study, abdominal distention. as well as. increasing abdominal girth (>2 cm) were used as signs of feeding intolerance. In relation to the above we must take into account that during nCPAP support, extremely preterm neonates present with benign abdominal distention (“CPAP belly syndrome”) in a percentage ranging from 45% [33] up to 90% [34] and in this case, abdominal distention is not a reliable indicator of feeding intolerance.

U/S is a useful method for the evaluation of gastric emptying in different clinical situations in preterm infants [35]. In the present study, the use of U/S allowed us to monitor milk clearance from the stomach over a 2-h period during both intermittent and continuous feeding methods. It is clear that ACSA measurements in intermittent-bolus feeding were significantly higher in the first 30 min, a difference that no longer existed at 60 min, and was subsequently reversed, with ACSA measurements becoming significantly higher in the continuously fed group at the 90 min and especially 120 min mark.

There are limitations in the current study. The most important limitation is the small sample size. Another limitation is that the gastric emptying time in continuously fed neonates was not successfully measured in all cases. Nonetheless, the strength of this study derives from the fact that the newborns were acting as controls of themselves and were fed with the same quantity and type of milk during both intervention periods (bolus and continuous feeding). Moreover, for each newborn, gastric volume changes based on ACSA measurements were serially evaluated during a 2-h period of U/S assessment.

## 5. Conclusions

In very preterm neonates, gastric emptying seems to depend on the mode of feeding. Our study showed that in neonates fed with the continuous feeding mode, no gastrointestinal complications were noted despite the fact that gastric residual volume based on ACSA values calculated reached up to 80% of gastric feeding volume. Thus, during continuous feeding, gastric residual volumes up to 80% could be acceptable. Further studies are necessary to verify our findings, as establishing an acceptable gastric residual in continuous feeding may justify changes in nutritional practices for this very susceptible population.

## Figures and Tables

**Table 1:** Baseline characteristics of the neonates study.

Number of neonates included	31
Males/Females	14/17
BW g (mean, SD)	982 (28)
GA weeks (mean, SD)	28 (1.5)
SGA (n)	7/31
Antepartum corticosteroids	19/31
1 min Apgar Score (median, range)	6 (1–9)
5 min Apgar Score (median, range)	8 (2–9)
Inborns/outborns (n)	15/16
RDS (n)	29
BPD (n)	19
Respiratory support during measurements (n, %)	
nCPAP	18 (58.06)
O_2_ < 30%	7 (22.58)
room air	6 (19.35)
Full enteral feeding days (median, range)	12 (10–21)
First measurement (days/median, range)	15.5 (11–32)
Second measurement (days/median, range)	19 (14–35)
Milk volume (mL/2 h) during the assessments (median, range)	17 (9–22)
Breast milk + suppl/formula (n)	21/10

Abbreviations: BPD, bronchopulmonary dysplasia; BW, birth weight; GA, gestational age; nCPAP, nasal continuous positive airway pressure; RDS, respiratory distress syndrome.

**Table 2:** Distribution of ACSA values (median/1st–3rd Quartile) according to feeding method (bolus vs. continuous) in the ultrasound (U/S) assessment time points with the corresponding *p* value.

	ACSA (cm^2^)		
Assessment Time after the End of Milk Administration	Bolus Feeding	Continuous Feeding	*p*-Value
0′	1.9 (1.4–3.6)	1.0 (0.6–1.7)	<0.001
10′	1.8 (1.5–3.0)	1.0 (0.7–1.7)	<0.001
20′	1.5 (1.2–2.5)	0.9 (0.6–1.7)	<0.001
30′	1.3 (1.0–2.0)	0.8 (0.5–1.8)	<0.001
60′	1.0 (0.5–1.0)	0.8 (0.5–1.8)	0.328
90′	0.4 (0.2–0.6)	0.7 (0.4–1.4)	<0.001
120′	0.1 (0.0–0.2)	0.8 (0.5–1.95)	<0.001

Abbreviations: on antral cross-sectional area (ACSA).

## Data Availability

The data presented in this study are available on request from the corresponding author.
